# Evaluation of the Efficacy and Accuracy of Super-Flexible Three-Dimensional Heart Models of Congenital Heart Disease Made via Stereolithography Printing and Vacuum Casting: A Multicenter Clinical Trial

**DOI:** 10.3390/jcdd11120387

**Published:** 2024-12-03

**Authors:** Isao Shiraishi, Masaaki Yamagishi, Takaya Hoashi, Yoshiaki Kato, Shigemitsu Iwai, Hajime Ichikawa, Tatsuya Nishii, Hiroyuki Yamagishi, Satoshi Yasukochi, Masaaki Kawada, Takaaki Suzuki, Takeshi Shinkawa, Naoki Yoshimura, Ryo Inuzuka, Yasutaka Hirata, Keiichi Hirose, Akio Ikai, Kisaburo Sakamoto, Yasuhiro Kotani, Shingo Kasahara, Toshiaki Hisada, Kenichi Kurosaki

**Affiliations:** 1 Department of Pediatric Cardiology, National Cerebral and Cardiovascular Center, Suita 564-8565, Japan; 2 Department of Pediatric Cardiovascular Surgery, Kyoto Prefectural University of Medicine, Kyoto 602-8566, Japan; 3 Department of Pediatric Cardiac Surgery, National Cerebral and Cardiovascular Center, Suita 564-8565, Japan; 4 Department of Pediatric Cardiac Surgery, Saitama Medical University International Medical Center, Hidaka 350-1298, Japan; 5 Department of Radiology, National Cerebral and Cardiovascular Center, Suita 564-8565, Japan; 6 Department of Pediatrics, Keio University School of Medicine, Tokyo 160-8582, Japan; 7 Heart Center, Nagano Children’ Hospital, Azumino 399-8288, Japan; 8 Division of Pediatric and Congenital Cardiovascular Surgery, Jichi Children’s Medical Center Tochigi, Shimotsuke 329-0498, Japan; 9 Department of Cardiovascular Surgery, Tokyo Women’s Medical University, Tokyo 162-8666, Japan; 10 Department of Thoracic and Cardiovascular Surgery, Graduate School of Medicine, University of Toyama, Toyama 930-0194, Japan; 11 Department of Pediatrics, The University of Tokyo, Tokyo 113-8655, Japan; 12 Department of Cardiovascular Surgery, The University of Tokyo, Tokyo 113-8655, Japan; 13 Department of Cardiovascular Surgery, Mt. Fuji Shizuoka Children’s Hospital, Shizuoka 420-8660, Japan; 14 Department of Cardiovascular Surgery, Okayama University Graduate School of Medicine, Dentistry, and Pharmaceutical Sciences and Okayama University Hospital, Okayama 700-8558, Japan; 15 Graduate School of Frontier Sciences, The University of Tokyo, Kashiwa 227-0871, Japan

**Keywords:** congenital heart disease, heart surgery, simulation, 3D printing, stereolithography, vacuum casting, clinical trial

## Abstract

Three-dimensional (3D) printing is an advanced technology for accurately understanding anatomy and supporting the successful surgical management of complex congenital heart disease (CHD). We aimed to evaluate whether our super-flexible 3D heart models could facilitate preoperative decision-making and surgical simulation for complex CHD. The super-flexible heart models were fabricated by stereolithography 3D printing of the internal and external contours of the heart from cardiac computed tomography (CT) data, followed by vacuum casting with a polyurethane material similar in elasticity to a child’s heart. Nineteen pediatric patients with complex CHD were enrolled (median age, 10 months). The primary endpoint was defined as the percentage of patients rated as “essential” on the surgeons’ postoperative 5-point Likert scale. The accuracy of the models was validated by a non-destructive method using industrial CT. The super-flexible heart models allowed detailed anatomical diagnosis and simulated surgery with incisions and sutures. Thirteen patients (68.4%) were classified as “essential” by the primary surgeons after surgery, with a 95% confidence interval of 43.4–87.4%, meeting the primary endpoint. The product error within 90% of the total external and internal surfaces was 0.54 ± 0.21 mm. The super-flexible 3D heart models are accurate, reliable, and useful tools to assist surgeons in decision-making and allow for preoperative simulation in CHD.

## 1. Introduction

Recent advances in diagnosis, management, and surgical techniques have significantly improved the survival and prognosis of patients with congenital heart disease (CHD) [1,2]. However, in some patients with complex CHD, the prognosis after the surgery remains unsatisfactory due to the anatomical and hemodynamic complexity of the disease [3,4,5]. Therefore, an accurate understanding of the heart structure and the subsequent appropriate design of the surgical procedures are critical to patient survival and better quality of life. 

Three-dimensional (3D) diagnostic modalities, such as cardiac computed tomography (CT), magnetic resonance imaging (MRI), and 3D echocardiography, have facilitated the diagnosis and surgical planning of complex CHD. For example, 3D images have shown the spatial relationships of the ventricles, valves, arteries, veins, trachea, and bronchi that are helpful to cardiovascular surgeons [6,7]. However, there can be discrepancies between the 3D images displayed on the flat screen and the real 3D structures of the heart. Therefore, the creation of tangible 3D heart models with similar configurations and textures would lead to significant improvements and innovations in CHD diagnosis and surgery.

The usefulness of 3D-printed heart models in the diagnosis and treatment of CHD has been widely reported and reviewed [8,9,10,11,12,13,14,15,16]. The quality of 3D-printed heart models has improved over the years, and 3D heart models have been recognized as a necessary tool for medical education and training in pediatric cardiac surgery [11,17]. To further advance the practical application of 3D printing technology, and to perform feasible simulated surgery with cutting and suturing similar to that of real heart tissue, we developed a unique technology to fabricate extremely flexible polyurethane-based 3D heart models using stereolithography 3D printing molds, followed by vacuum casting. The combination of these technologies allows the reproduction of 3D models with different types of elasticity and flexibility. The main purpose of developing and manufacturing the super-flexible 3D models is that these patient-specific 3D heart models will enable surgeons to perform preoperative simulation surgery with cutting and suturing, which would improve the outcome of surgery and the prognosis of patients. The usefulness of the super-flexible 3D models was evaluated in a retrospective study of 20 consecutive cases of complex CHD [18]. The purpose of this study was to conduct a physician-led clinical trial to further confirm the efficacy and safety of the super-flexible 3D heart models and to obtain their approval as an official medical device.

## 2. Materials and Methods

### 2.1. Study Design and Patients

**Figure 1:**
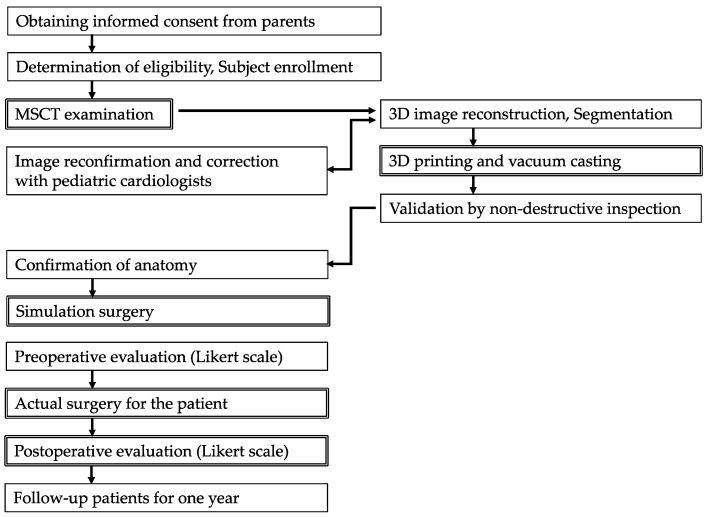
Flowchart of the fabrication and evaluation of the super-flexible 3D heart models. The left side of the diagram shows the process used by the clinical institutes, and the right side shows the process used by the manufacturer.

The flowchart of the fabrication and evaluation of the super-flexible 3D heart models is shown in Figure 1. This clinical trial was a single-arm, open-label, multicenter (five representative pediatric cardiology institutes in Japan), prospective validation study of the super-flexible 3D heart models. The study was conducted in accordance with relevant guidelines and in compliance with the Declaration of Helsinki. This clinical trial was approved by the Institutional Review Board of the National Cerebral and Cardiovascular Center (IRB1118, 23 December 2019) and was registered in the Japan Registry of Clinical Trials (jRCT 2052200093, 10 December 2020). The protocol of the 1-year follow-up study was also approved by the Research Ethics Committee of the National Cerebral and Cardiovascular Center (R20107, 22 February 2021).

The inclusion criteria for the study were as follows:

1. Pediatric patients (age < 15 years) with complex CHD requiring open heart surgery for their life support.
2. Patients with complex 3D heart structures and great vessels, where morphological diagnosis and preoperative simulation are considered essential.
3. Patients requiring cardiac CT angiography and cardiac catheterization for diagnosis and surgical strategy.
4. Complex CHD diagnoses, including (1) double outlet right ventricle (DORV), (2) transposition of the great arteries (TGA), (3) congenitally corrected transposition of the great arteries (cc-TGA), (4) multiple ventricular septal defects (mVSD), (5) functionally univentricular heart (UVH), including unbalanced atrioventricular septal defect (AVSD), (6) tetralogy of Fallot (TOF) with pulmonary atresia or severe right ventricular (RV) outflow tract stenosis, and (7) hypoplastic left heart syndrome and its related anomalies (HLHS).

Exclusion criteria were as follows:

1. Patients requiring emergent or semi-emergent surgery before completion of the super-flexible 3D heart models.
2. Patients ineligible for this clinical trial.

### 2.2. CT Image Acquisition, 3D Reconstruction, and Segmentation

Cardiac CT angiography (SOMATOM Force, SOMATOM Sensation 64, SOMATOM Definition Flash, Siemens, Munich, Germany, or Aquilion ONE, Canon, Tokyo, Japan) was performed under oral sedation with triclofos sodium and/or intravenous injection of midazolam or thiopental. A 1.0–2.0 mL/kg bolus of contrast medium (180–370 mg iodine/mL) was injected into the peripheral veins at the rate of 0.5–1.0 mL/kg with a 10–20 mL saline flush with the same injection rate using a dual-head injector. After 15–18 s of injection, the CT angiography was started with a median tube voltage and current of 70 kV and 44 mAs, respectively. Image acquisition was performed with or without ECG (electrocardiography) gating, depending on the patient’s heart rate. Breath-holding was not used because most patients were infants and toddlers. Subsequently, 3D volumetric image data were reconstructed with a slice thickness of 0.25–1.0 mm.

The 3D data were transferred to the 3D model manufacturing company for the following procedures. Segmentation of the endocardial contour of the atrium, ventricle, and vessel was performed using the thresholding method, where the contrast-enhanced heart and vessel lumen were delineated by searching the appropriate threshold Hounsfield unit (HU) according to the patient’s anatomical features (Mimics, Materialize Inc., Leuven, Belgium). The epicardial contour of the ventricles and atria was determined by fitting the HU to the epicardial boundaries of each ventricle and atrium. The ventricles were sometimes delineated separately because the HUs of the left and right ventricular myocardium differed according to the coronary blood flow. The outer boundaries of the vessels were delineated within 1.0–1.5 mm of the vessel contour, depending on the patient’s age and body weight. Final volume rendering (VR) images of the whole heart were obtained by merging and aligning the images of the atria, ventricles, and vessels using 3D digital modeling software (Geomagic Freeform Plus, 3D Systems, Rock Hills, SC, USA). To minimize image reconstruction artifacts, the attending pediatric cardiologists carefully reviewed and modified the VR images after segmentation based on the echocardiographic and angiographic findings. The final images were always reviewed by the surgeons and improved if necessary.

### 2.3. Chemical Composition and Mechanical Properties of the Polyurethane Material

The material used for the 3D heart models was a 100% polyurethane with a texture similar to human skin, HaplaPuddingGel (Polysis Co., Ltd., Hamamatsu, Japan) [19]. The chemical compositions are registered as a Japanese patent (Patent No.: 6309664, Patent holder: Polysis Co., Ltd.).

The physical properties of actual polyurethane used were investigated by tensile tests at 100% (λ = 2) and 300% (λ = 4) of strain. Young’s modulus was evaluated using linear and quadratic approximations of E_11_ ((1,1) component of the tensor) and S (nominal stress).

### 2.4. Stereolithography 3D Printing and Vacuum Casting

**Figure 2:**
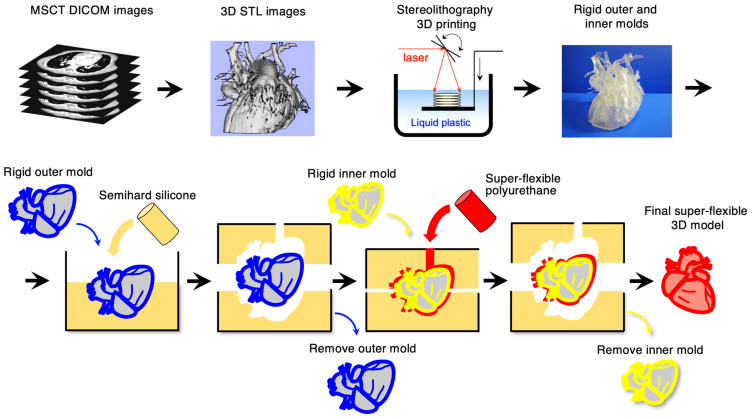
Fabrication of full-scale and flexible 3D heart models. The (**top panel**) shows the image acquisition, segmentation, and stereolithography 3D printing process. The (**bottom panel**) shows the vacuum casting process using a silicone mold.

The fabrication processes of the super-flexible 3D heart model [18] are shown in Figure 1 (top panel: stereolithography 3D printing, and bottom panel: vacuum casting). The segmented 3D data formatted in Digital Imaging and Communication in Medicine (DICOM) were converted into standard triangulated language (STL) files using commercially available software (Mimics, Materialize Inc., Belgium) and stored. The STL data of the external and internal heart contours were used to guide the ultraviolet laser beam to polymerize a selectively photosensitive liquid polymer solution, known as stereolithography 3D printing (Figure 2 top panel, SOUP2 600GS, CMET, Yokohama, Japan; SCS-8100, Sony Manufacturing Systems Inc., Tokyo, Japan).

The stereolithography plastic model of the external heart contour was used to create the external silicone mold for vacuum casting. After solidification, the external silicone mold was cut, and the plastic model was removed. The stereolithography model of the internal heart contour was then carefully inserted into the external silicone mold. After precisely adjusting the direction and position, the polyurethane material for the final super-flexible 3D heart models (physical properties are shown in Section 3.1) was injected between the external and internal molds under vacuum conditions (Figure 2 bottom panel, SOV-200, Nippon Sosei, Kogyo, Japan). After the polyurethane material solidified, the external silicone mold was carefully cut and removed, and the polyurethane 3D model of the heart was obtained. The stereolithographic internal mold was crushed and removed. Subsequently, the final super-flexible 3D heart models were obtained. Magenta- or cream-colored 3D heart models were reproduced according to the surgeons’ preference.

The total time to produce the super-flexible 3D heart models (from receiving the DICOM data to shipping the final product) was 8.8 ± 3.4 days (mean ± standard deviation [S.D.]). The super-flexible 3D heart models cost USD 2000 to 3000 per model, depending on the size and the total time required. 

### 2.5. Error Validation of Stereolithography and Vacuum Casting

To validate the accuracy and reproducibility of the 3D heart models, the final products were scanned using industrial CT for non-destructive inspection (METROTOM 800, Carl Zeiss, Oberkochen, Germany). To stabilize the super-flexible 3D heart models during CT scanning, the support materials of the 3D heart models––models of the heart and great vessels––were designed separately by computer-aided design based on the original volume rendering data after segmentation and manufactured from Styrofoam. The 3D heart models were fixed with the Styrofoam support materials and scanned with CT.

The volumetric data of the final products were compared with the original patient 3D data for stereolithography using 3D image validation software (VGSTUDIO MAX3.0, Volume Graphics, Heidelberg, Germany). Discrepancies between the two sets of data were presented as a pseudocolor display.

### 2.6. Clinical Evaluation of the 3D Heart Models

To quantify the usefulness of the models, anatomical checkpoint scoring charts for CHD were created for eight CHDs (DORV, n = 16; TGA, n = 16; cc-TGA, n = 16; CoA/IAA, n = 4; mVSDs, n = 4; UVH, n = 3; TOF, n = 10; and HLHS, n = 4). After the real surgery, the primary surgeons rated the efficacy of the 3D models based on a 5-point Likert scale [20].

After preoperative morphologic diagnosis and surgical simulation, the surgeons rated the effectiveness of the super-flexible 3D heart models based on a 5-point Likert scale (“essential”, “very useful”, “useful”, “not useful”, or “misleading”) for each anatomical target site. Pre- and postoperative total scores were determined based on the combined anatomical checkpoints and simulation scores.

The 5-point Likert scale used in this study had the following score categories:

- Essential: Without additional information from the 3D models, it was not possible to make appropriate preoperative plans for the patient.
- Very useful: Preoperative planning with the 3D models provided additional information to that provided by existing diagnostic devices.
- Useful: The 3D models provided useful information for preoperative planning, which slightly differed from that provided by existing diagnostic devices.
- Not useful: The 3D models had uncertain imaging information and were unsuitable for preoperative planning.
- Misleading: The 3D models had inappropriate information that misled the preoperative planning process.

### 2.7. Endpoints and the Basis for Adjustment

The primary endpoint was the percentage of cases rated as “essential” on the 5-point Likert scale by the primary surgeons. The criteria for determining device usefulness were set such that the lower bound of the 95% confidence interval (CI) for the percentage of cases deemed “essential” was ≥30%. The basis for determining the primary endpoint was as follows: the device was designed to improve the accuracy of the morphological diagnosis of CHD. If the accuracy is improved, a more appropriate surgical procedure can be selected, considering the patient’s good quality of life. Therefore, the usefulness of the models would be measured by how the morphological and procedural information obtained from the 3D heart models was reflected in the actual surgery and how beneficial it was.

On the Likert scale used to rate the usefulness of the super-flexible 3D heart models, “essential” was defined as when the surgery could not have been performed appropriately without the additional information provided. Based on this, the primary endpoint was the percentage of cases deemed “essential” by the primary surgeon’s postoperative evaluation. Regarding the validity of the 30% benefit threshold, it was believed that performing hemodynamically appropriate surgery for the “essential” cases would have been challenging without the device and their success would have been unlikely. The difference between successful and unsuccessful cases with appropriate surgery was clear. Therefore, even if the benefit rate was 10%, it was significant, and the expected benefit rate in this clinical trial was set to at least 30% based on the results of the previous study [18].

The secondary endpoints were as follows: (1) a percentage of patients overall rated as “essential” (validity assessment by the efficacy evaluation committee), (2) a percentage of patients overall rated as “essential” or “very useful” (postoperative assessment by the primary surgeons), and (3) a percentage of cases overall rated as “essential” or “very useful” (postoperative assessment by the efficacy assessment committee).

### 2.8. External Committees and Follow-Up

To confirm the validity of the primary surgeon’s assessment, an external efficacy assessment committee consisting of three experienced pediatric cardiac surgeons independent of this clinical trial was established. The procedures of the committee were (1) validation of the diagnosis and planned surgical procedures before receipt of the 3D heart models, (2) modification of the preoperative procedure and validation of the assumed assessment after the receipt of the 3D heart models, and (3) postoperative modification of the surgical procedure and validity of the overall assessment.

To validate the post-simulation adverse events, a safety assessment committee consisting of three experienced pediatric cardiologists or cardiac surgeons was established.

Patients were followed up for one year after cardiac surgery. Adverse events, reoperations, catheter interventions, developmental or neurological problems, and life expectancy were evaluated.

### 2.9. Data Analysis

Data were collected, stored, and analyzed using Microsoft Excel (version 16, Microsoft, Redmond, WA, USA) and SAS Viya (SAS Institute Inc., Cary, NC, USA).

## 3. Results

### 3.1. Physical Properties of the Polyurethane Material

The results of tensile tests on three polyurethane samples were as follows: at a stretch of 2.0 (λ = 2.0), the nominal stress (S_N_) values were 93, 95, and 94 KPa; at λ = 4.0, the S_N_ values were 547, 497, and 452 KPa. The room temperature was 23 ± 2 °C. Because the Green–Lagrange strain tensor is commonly used for large strain analysis, the stretch λ was converted to the uniaxial form of the Green–Lagrange strain as follows:

E_GL_ = (λ^2^ − 1)/2

The calculated E_GL_ values for λ = 2 and 4 were 1.5 and 7.5, respectively. With these six stress–strain data points, we assumed linear and quadratic constitutive equations and calculated their coefficients by the least squares method. The resulting constitutive equations are as follows:

S_N_ = 65.1E_GL_

S_N_ = 61.9E_GL_ + 0.778E_GL_^2^

**Figure 3:**
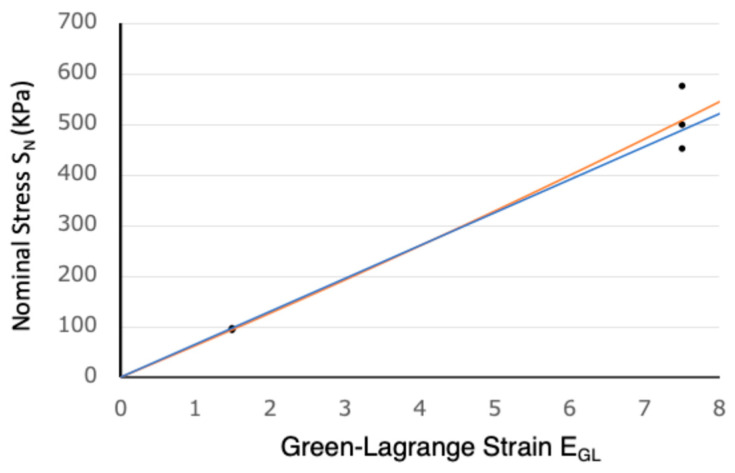
The measured stress–strain data points and the linear (blue) and the quadratic (orange) approximations.

These equations were plotted with six data points and showed a strong linearity between S_N_ and E_GL_ (Figure 3). The slope at the origin (61.9~65.1 KPa) is compared to Young’s modulus of normal pediatric heart tissue [21] in Section 4.1.

### 3.2. Patient Characteristics

Informed consent was obtained from 22 pediatric patients with complex CHD. Two patients were withdrawn from the clinical trial prior to surgery. One patient with a DORV with subpulmonary VSD required emergent coarctation repair with bilateral pulmonary artery banding. In the other patient with TOF and complete atrioventricular septal defect, surgery was postponed for over 90 days owing to the patient’s unfavorable general condition. The full analysis set was defined as 20 participants, excluding these two patients. The per-protocol set consisted of 19 participants, excluding one patient who was deemed by the efficacy assessment committee not to meet the inclusion criteria of “CHD patients with the extremely complex 3D structure of the heart and great vessels”. Table 1 shows the demographics of the 19 patients. Six patients had DORV, four had TOF (with pulmonary atresia or severe stenosis), four had HLHS and associated conditions, three had UVH, and two had TGA. There were 10 males and 9 females. The median age of the 19 patients was 10 months. The mean body weight was 7.7 ± 4.2 kg (mean ± S.D.).

### 3.3. Error Validation of Stereolithography and Vacuum Casting

**Figure 4:**
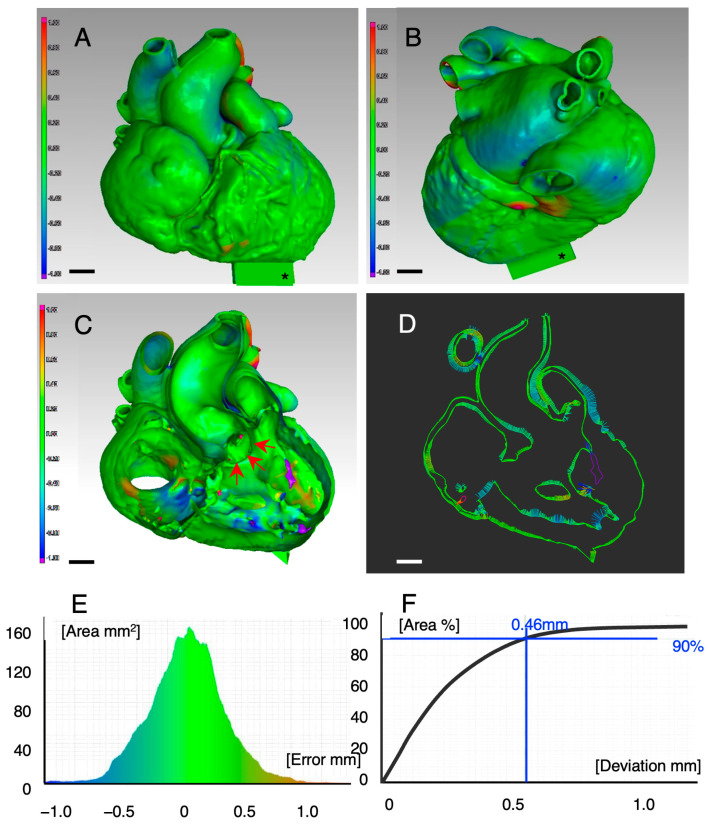
Error validation of stereolithography and vacuum casting (Case #2). Errors in the external (**A**,**B**) and internal (**C**) surfaces are shown as a pseudocolor representation (arrows indicate the edge of the VSD). (**D**): Vector plot on the split surface of (**C**). (**E**): Histogram of the errors. (**F**): The deviation curve of the total surfaces. VSD: ventricular septal defect. *: identification mark.

Figure 4 shows the pseudocolor representation of the external (A, anterior and B, posterior) and internal (C) surfaces of Case #2 (TOF with severe right ventricular outflow stenosis). Figure 4D shows a vector display on the cross-section of Figure 4C. Figure 4E shows a histogram of the errors, including those of the external and internal surfaces. Figure 4F shows the total area deviation curve. In Figure 4A,B, almost all external surfaces were green, indicating that the errors were within 0.3 mm. In Figure 4C, several blue and purple areas are at the right ventricular trabeculation and the bottom of the right atrium, indicating that the errors in these areas were above 0.6 mm. However, the VSD edge (arrows) was green, indicating that its size and location were accurately reproduced in the super-flexible 3D heart model. A histogram of the errors, including those of the external and internal surfaces, shows a symmetric normal distribution (Figure 4E). The error in 90% of the area was 0.46 mm (Figure 4F). The calculated mean error of the 19 patients, including those of the external and internal surfaces, was 0.58 ± 0.23 mm (mean ± S.D.), which is considered within the acceptable error range for CHD surgery.

### 3.4. Anatomical Diagnosis Using Super-Flexible 3D Heart Models

**Figure 5:**
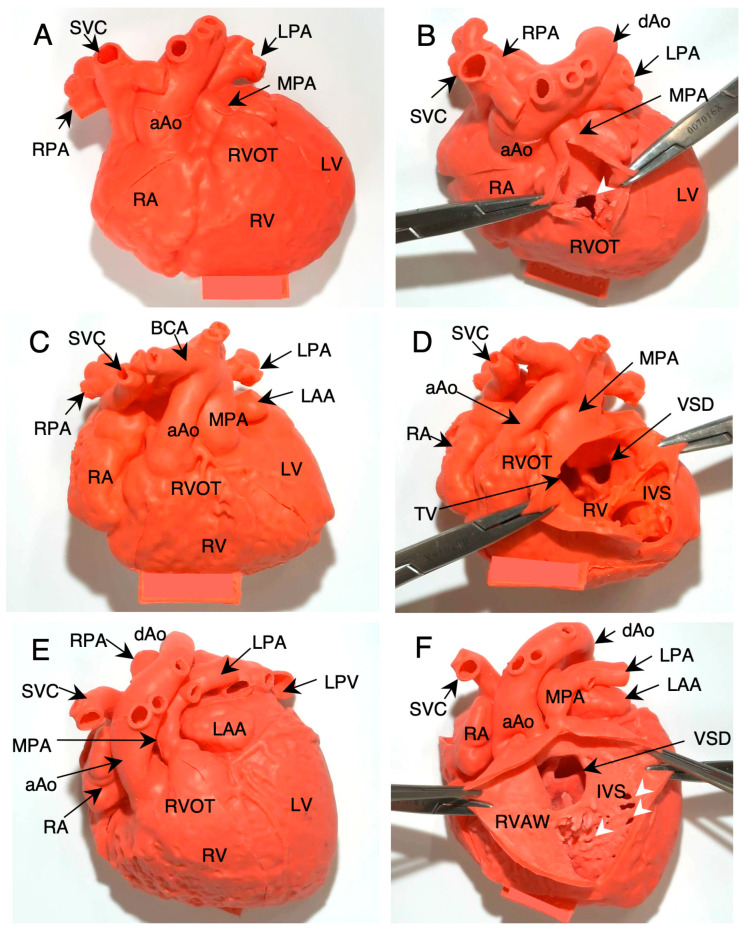
Anatomical diagnosis using super-flexible 3D heart models. (**A**,**B**): Nine-month-old male infant with tetralogy of Fallot (Case #1). The white arrow indicates severe right ventricular outflow obstruction. (**C**,**D**): A 6-month-old female infant with DORV (non-committed VSD) and interrupted aortic arch (Case #8). (**E**,**F**): An 18-month-old female infant with right isomeric heart, large VSD, pulmonary stenosis, and total anomalous pulmonary venous drainage (Case #10). Arrowheads indicate small muscular VSDs, VSD: ventricular septal defect, TA: tricuspid annulus, RVAW: right ventricular anterior wall.

Figure 5A,B show a super-flexible 3D model of a 9-month-old male infant with TOF (Case #1). The arrowhead in Figure 5B indicates RV outflow tract stenosis.

Figure 5C,D show the 3D model of a 6-month-old female infant with DORV (non-committed VSD) and hypoplastic aortic arch owing to subaortic stenosis, status post Norwood procedure with BT-shunt (Case #8). The ascending aorta lies in the right anterior portion of the main pulmonary artery (Figure 5C). The aorta and pulmonary artery originate from the right ventricle (Figure 5D).

Figure 5E,F show an 18-month-old female infant with a right isomeric heart, large VSD, pulmonary stenosis, and total anomalous pulmonary venous return (TAPVR), status post TAPVR repair with main pulmonary artery banding (Case #10). The ascending aorta lies in the right anterior portion of the main pulmonary artery (Figure 5E). A large and non-committed VSD is seen in the muscular inlet to the trabecular portion of the IVS (Figure 5F). Small and multiple honeycomb VSDs are seen in the apical portion of the IVS (arrows in Figure 5F). Hence, the entire IVS is thin and overall hypoplastic.

Overall, valve tissue could not be reproduced due to the rapid heartbeat of the children and the time resolution of the cardiac CT scanner. However, the attachment sites of the valves could be recognized as small protrusions, and we were able to distinguish subvalvular from supravalvular tissue by these protrusions and sinuses of Valsalva. Superimposing 3D echocardiographic reconstruction on CT images would address this problem [22].

### 3.5. Simulation Surgery

#### 3.5.1. Norwood Procedure for HLHS with Chimney Reconstruction

**Figure 6:**
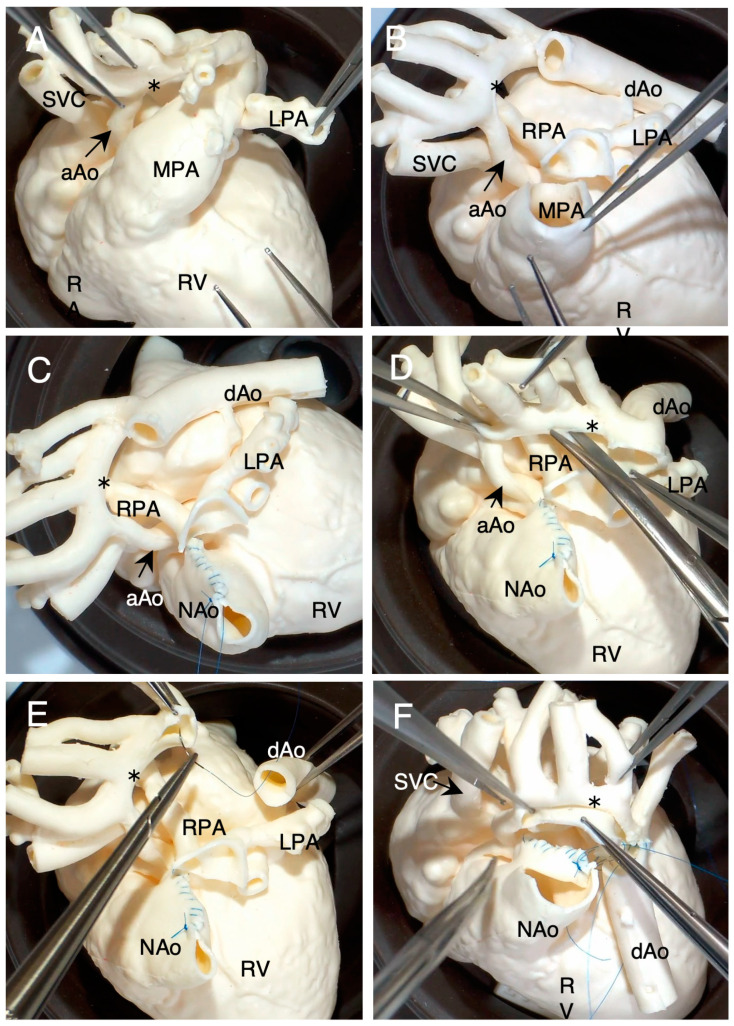
Simulated Norwood procedure (chimney reconstruction of the aortic arch [18,19] in a 3-month-old infant with hypoplastic left heart syndrome (Case #15)). Asterisks in (**A**–**F**) indicate the hypoplastic aortic arch. SVC: superior vena cava, aAo: ascending aorta, dAo: descending aorta, NAo: neoaorta, MPA: main pulmonary artery, LPA: left pulmonary artery, RPA: right pulmonary artery, RA: right atrium, RV: right ventricle.

Figure 6 shows a simulated Norwood procedure (chimney reconstruction of the aortic arch [23,24]) on a 3-month-old infant with HLHS after bilateral pulmonary artery banding (Case #15). Figure 6A shows the top view of the 3D model before simulation. After resection of the ductal tissue (Figure 6B), the pulmonary artery orifices were excised as a U-shaped cuff from the main pulmonary artery (MPA, Figure 6B). The posterior U-shaped defect of the MPA was sutured longitudinally to plicate it to create a chimney-like neoaorta (Figure 6C). Subsequently, the lesser curvature of the aortic arch was incised (Figure 6D), and the remaining ductal tissue was completely resected (Figure 6E). The neoaorta was partially incised and anastomosed to the aortic arch (Figure 6F). The super-flexible 3D heart model was used to determine the relationship between the hypoplastic aortic arch and the main pulmonary artery. In the postoperative review by the primary surgeon, the 3D heart model was rated as “very useful” in determining the incision line of the hypoplastic aortic arch, determining the anastomosis line between the PT and the aortic arch, and understanding the left and right pulmonary arteries after resection.

#### 3.5.2. Half-Turned Truncal Switch Operation in TGA with Severe Pulmonary Stenosis

**Figure 7:**
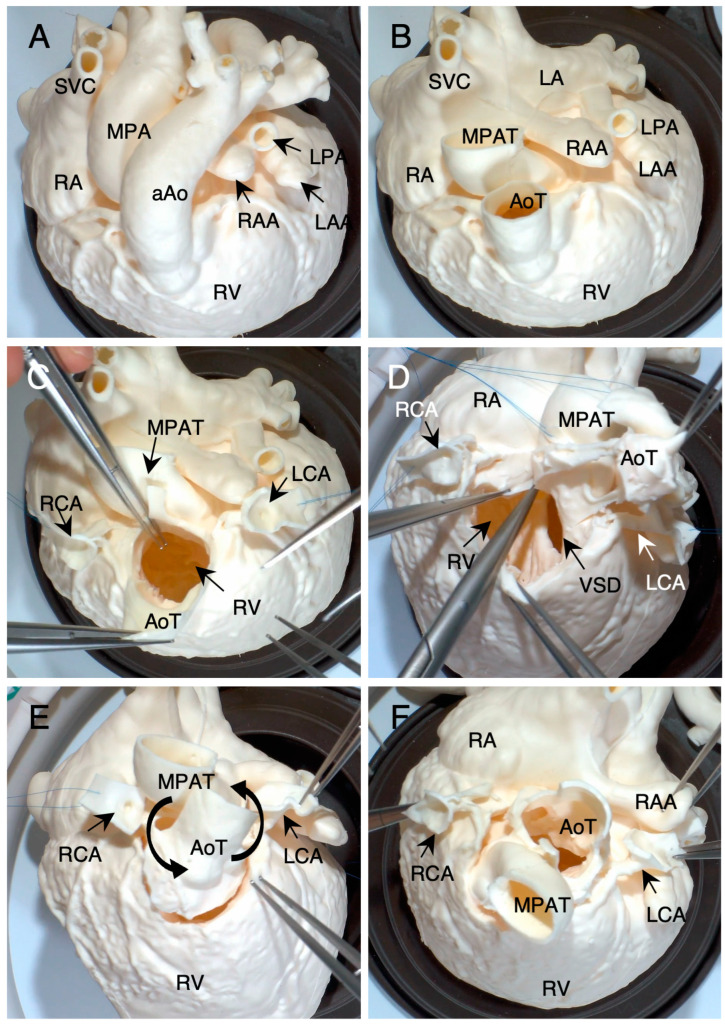
(**A**–**F**) Simulated surgery of the half-turned truncal switch procedure [20,21] in a 15-month-old infant with transposition of the great arteries and severe pulmonary stenosis (Case #12). Abbreviations are shown in Figure 6. RAA: right atrial appendage, LAA: left atrial appendage, LA: left atrium, RCA: right coronary artery, LCA: left coronary artery, MPAT: main pulmonary arterial trunk, AoT: aortic trunk, VSD: ventricular septal defect.

Figure 7 shows a simulated half-turned truncal switch operation [25,26] on a 15-month-old infant with TGA with severe pulmonary stenosis (Case #15). Figure 7A shows the top view of the super-flexible 3D heart model before simulation surgery. The ascending aorta and the main pulmonary artery were initially transected above the coronary artery orifices (Figure 7B). Both coronary artery buttons were resected similar to the arterial switch operation (Figure 7C). Subsequently, the aortic and main pulmonary artery were resected en bloc from the ventricular outflow tract as a truncal block (Figure 7D). The resected truncal block was half-turned and anastomosed to the ventricular outflow tract (Figure 7E,F). In the postoperative review by the primary surgeon, the super-flexible 3D heart model was rated “essential” for determining the truncal block resection line, VSD closure suture line, VSD patch design, and availability of coronary artery translocation. Simulated surgeries were performed using polytetrafluoroethylene (ePTFE) patches or conduits, as in real surgery for patients.

### 3.6. Anatomical Checkpoints and Overall Scores

The average scores at each anatomical site are shown as bar graphs in Supplementary Figure S1 (n = 4 in TOF, n = 6 in DORV, n = 2 in TGA, n = 4 in HLHS, n = 3 in UVH). The anatomical scoring table quantified “essential” as a score of 4, “very useful” as a score of 3, “useful” as a score of 2, “not useful” as a score of 1, and “misleading” as a score of 0. The scores were high (>3.0) for the evaluation of the left and right ventricular outflow tracts, the shape and margin of the VSD in DORV, the incision sites of the great arteries in TGA, the morphology and size of the RV in TOF, the origin and course of the great arteries and veins, and the morphology of the bilateral pulmonary arteries in UVH.

The results of the planned and actual surgeries after simulation, overall Likert scale scores, and 1-year follow-up are shown in Table 2. The results of the endpoints are summarized in Table 3. The primary endpoint was set as the proportion of cases with an overall rating of “essential” on the 5-point Likert scale (postoperative rating by the primary surgeons). Thirteen of the 19 patients were rated as “essential” by the primary surgeons. The efficacy rate was 68.4%, and the 95% CI was 43.4–87.4%, indicating that the results met the primary endpoint (the lower limit of the CI was greater than 30%).

In the secondary endpoint analysis, the surgeon rated 17 of the 19 patients as “essential” or “very useful” (the efficacy rate was 89.5%, and the 95% CI was 66.9–98.7%). The efficacy assessment committee rated 11 of the 19 patients as “essential” after surgery (the efficacy rate was 57.9%, and the 95% CI was 33.5–79.7%). Similarly, they rated 17 of the 19 patients as “essential” or “very useful” (the efficacy rate was 89.5%, and the 95% CI was 66.9–98.7%). The overall lower limit of the CI was >30%, meeting the secondary endpoint (Table 3).

The mean operative time for cardiac arrest was 105.4 ± 61.9 (0–239) minutes, and the mean intensive care unit (ICU) stay of the 19 patients was 13.0 ± 9.7 (2–30) days. Four patients (21.0%) required a cardiopulmonary bypass in the ICU for a mean period of 93.0 ± 41.6 h. Within 30 days postoperatively, three of the 19 patients underwent additional surgeries (15.8%, mediastinal hemorrhage, aortic arch reconstruction, and pericardial effusion). No catheter interventions were performed during the same period.

### 3.7. Changes in Surgical Procedures After Simulation

Four participants (21.1%) underwent different surgical procedures after simulation with the super-flexible 3D heart models (Table 2). In the first patient (Case #10), diagnosed with DORV with a large VSD, the initial biventricular repair (Rastelli procedure) was changed to a single ventricle physiology repair (bidirectional Glenn anastomosis) because the 3D heart models showed multiple large VSDs that were difficult to close with the Rastelli procedure.

In the second patient (Case #7), diagnosed with DORV and non-committed VSD, the primary surgeon could not decide whether the biventricular or functionally univentricular repair was appropriate. After a detailed review of the super-flexible 3D heart models, the surgeon determined that the distance between the left ventricle and the aorta was excessively long to create a sufficient intraventricular routing. Hence, the surgeon selected the bidirectional Glenn anastomosis procedure. During the actual surgery, the distance between the left ventricle and aorta and the potential interference of the tricuspid valves with the interventricular pathway further confirmed that the single ventricular physiology repair would be appropriate.

In the third case (Case #11), the diagnosis was the complete transposition of the great arteries with small multiple muscular VSDs. The planned procedure was an arterial switch operation, and the VSDs were going to be left open because the multiple small VSDs were located at the apical portion of the ventricular septum and were challenging to close. However, after reviewing the 3D heart models, the surgeon determined that the VSDs could be closed using the sandwich technique. During the actual surgery, the multiple VSDs were successfully suture-closed.

In the fourth case (Case #2), the diagnosis was pulmonary atresia with ventricular septal defect. Right ventricular outflow reconstruction with a transannular patch was initially planned. However, the 3D heart model showed that a valve-sparing procedure was feasible. The surgeon performed the reconstruction without incising the right ventricular outflow muscle and pulmonary valve leaflets.

### 3.8. Safety Assessment and Follow-Up Study

There were no structural inconsistencies in the anatomical diagnosis between the super-flexible 3D heart models and the patients’ real hearts during chest opening. There were no malfunctions attributed to the 3D heart models during the clinical trial period. No adverse events were observed owing to malfunction of the 3D heart models.

Serious adverse events unrelated to the 3D heart models occurred in four cases (21.1%): systemic ventricular failure, hypotension owing to decreased pulmonary vascular resistance, cerebral infarction, and cardiac arrest. Similarly, 18 of the 19 patients were alive 30 days after surgery, for a survival rate of 94.7%. One patient primarily diagnosed with HLHS (Case #16) died of systemic ventricular failure 15 days after the surgery. Autopsy revealed a hypoplastic left main coronary artery and multiple organ anomalies, including an absence of the left kidney and a cleft palate and cleft lip. The safety assessment committee concluded that a causal relationship with the 3D heart models could not be established.

Two patients experienced serious adverse events between 1 month and 1 year of follow-up. One patient with HLHS (Case #13) died of multi-organ failure owing to bacteremia 87 days postoperatively. Another patient with a double outlet right ventricle after the Norwood procedure (Case #5) underwent aortic arch reconstruction 44 days after the initial procedure.

### 3.9. Application for Authorized Medical Device

After completion of the clinical trial and 1-year follow-up, the trial results were submitted to the Pharmaceuticals and Medical Devices Agency (PMDA) in Japan for approval as an authorized medical device. According to the suggestion of the PMDA, an adaptation guideline for the super-flexible 3D models for CHD was prepared in 2022 in collaboration with the Japanese Society of Pediatric Cardiology and Cardiac Surgery. The Japanese Ministry of Health, Labor, and Welfare finally approved the 3D flexible heart models as a Class II (low risk) medical device (30500BZX00177000) in July 2023. The devices are intended to diagnose cardiac structures in patients with complex CHD that are difficult to diagnose and determine surgical procedures using existing imaging modalities and are used in conjunction with other diagnostic modalities to assist in surgical planning.

## 4. Discussion

In this study, the research, development, and clinical trial of the super-flexible 3D heart model yielded three major results: (1) Our original technique of stereolithography 3D printing and vacuum casting enabled the production of 3D heart models with a precise size, shape, elasticity, and texture suitable for accurate anatomical diagnosis and preoperative simulation of complex CHD with cutting and suturing. (2) The multicenter clinical trial confirmed that the flexible 3D heart models were useful and safe to assist surgeons in the decision-making process, with no significant adverse events. (3) The super-flexible 3D heart model was finally approved as an authorized medical device.

### 4.1. Mechanical Properties of the Polyurethane Materials and Accuracy of the Models

The tensile tests were conducted for large strains such as E_GL_ = 1.5 and 7.5 (λ = 2 and 4), and, as shown in Figure 3, a strong linearity was found between S_N_ and E_GL_. Since the Green–Lagrange strain and the nominal stress are theoretically reduced to the infinitesimal strain (λ − 1) and the Cauchy stress (true stress), respectively, for a small strain range, the slope at the origin (61.9~65.1 KPa) may be regarded as the Young’s modulus.

Chaturvedi et. al. [21] reported that the Young’s modulus measured at a physiological small strain (λ = 1.05) of normal heart tissue was 59.1 KPa, and those of the volume-overloaded and pressure-overloaded heart tissues obtained from congenital heart disease ranged from 20.7 KPa to 145.6 KPa. Thus, the super-flexible 3D heart models have a stiffness similar to that of human heart tissue and would be suitable for simulation surgery involving cutting and suturing. Another characteristic of the polyurethane material used in this study is that it has a much better % elongation at break value (700%) [19] compared to other 3D printing materials (e.g., Stratasis Agilus30 at 220–270% [27] and Formlabs Silicone 40A resin at 170–220% [28]). This makes it suitable for simulated surgery, especially suturing.

Stereolithography is one of the most accurate and reliable 3D printing methods, and the layer pitch used was 0.1 mm. In this study, polyurethane materials with varying elasticity and flexibility were jointly molded using vacuum casting, enabling preoperative simulation. A concern is the defect caused by the molding process. For the first time, we used a non-destructive inspection method using an industrial CT scanner and geometry analysis software to validate 3D printing. The average surface error, including those of the outer and inner surfaces, between the volumetric data of the CT-scanned final products and those of the original patients after segmentation was 0.58 ± 0.23 mm. Considering that the spatial resolution of cardiac CT in this clinical trial was 0.3–0.5 mm depending on the institution and considering previously published reports using manual measurements and the manual skill of the surgical technique [29,30,31], the error in the super-flexible 3D heart models is likely to be acceptable to surgeons.

The primary reason for developing super-flexible 3D heart models with a complex manufacturing process is to realize patient-specific preoperative simulation with cutting and suturing using 3D models with texture and elasticity similar to a real heart. Recently, various types of silicone-based, flexible, 3D-printed heart models of CHD were developed [17,32,33,34]. These models, as well as our super-flexible polyurethane-based 3D heart models, offer excellent physical properties superior to traditional solid or semi-flexible 3D-printed heart models and are ideal tools for training and surgical simulation.

### 4.2. Clinical Trial

In this clinical trial, the overall assessment of “essential” was 68.4%, with a lower limit of the 95% CI of 43.4% (>30%), meeting the primary endpoint of the study. The results of this clinical trial indicate that the super-flexible 3D heart models provided surgeons with new medical information that could only be obtained by cutting and opening the heart cavity of the tangible and super-flexible 3D models. This is not to say that surgeons would not be able to perform appropriate surgical procedures for each patient without the 3D heart models, but that the new medical information provides significant and critical assistance in the decision-making about surgical procedures and techniques. Because the reasons for “essential” varied depending on the diagnosis, the cases of DORV with non-committed VSD, TGA with severe pulmonary stenosis, and functionally univentricular heart are discussed below.

The medical institutions that participated in this clinical trial are tertiary pediatric cardiology facilities with world-class expertise in CHD surgery, and all primary surgeons have extensive surgical experience. In this clinical trial, even the experienced surgeons agreed that the super-flexible 3D heart models were useful for preoperative decision-making and provided rational surgery. It is expected that the super-flexible 3D heart models will be more widely used for sharing 3D anatomy during preoperative conferences with surgical assistants and nurses, for technical training and teaching of surgical skills, and for explaining cardiac structures and surgical procedures to patients and parents. In fact, physician survey studies reported that a preoperative simulation using 3D-printed models improves surgeons’ hands-on skills and the possibility to reduce operation time [35,36,37,38,39].

In the case of DORV with non-committed VSD, determining whether biventricular repair is feasible using only conventional imaging modalities such as 2D echocardiography or angiography can be challenging. The 3D heart models allowed confirmation of the specific location and size of the VSD and the ability to secure the right ventricular cavity by creating an intraventricular pathway, thus allowing determination of the best surgical procedure [40]. In Case #7, the surgeon was unable to decide whether a biventricular or functionally univentricular repair was appropriate using conventional diagnostic methods alone. Careful observation of the ventricular cavities using the super-flexible 3D heart models revealed that the aorta originated further from the VSD than expected, and it was predicted that the tricuspid valve and its apparatus would be an obstacle to the intraventricular pathway. The creation of the intraventricular pathway proved to be technically challenging. The surgeon conducted a single ventricle repair and performed a Glenn anastomosis. In Case #10, the 3D models showed that the extensive defect of the ventricular septum, thinning of the remaining septum, and multiple small VSDs made the Rastelli-type procedure impossible. Therefore, the Glenn anastomosis was performed.

In TGA with severe pulmonary stenosis (Case #12), the surgeon chose to perform a half-turned truncal switch operation, avoiding the need for intraventricular reconstruction with an artificial conduit, based on the spatial relationships of the VSD, pulmonary arteries, and ascending aorta, using the super-flexible 3D heart models. The super-flexible 3D heart model also assisted the surgeon in determining the truncal block resection line, the VSD closure suture line, the VSD patch design, and the availability of coronary artery translocation. As a result, the procedure was finally assessed as “essential”.

All patients with a functionally univentricular heart associated with right or left isomeric heart had undergone Fontan surgery and required this experimental device to determine the conduit design for the procedure. In Case #17, the 3D heart model was used to determine the appropriate position of the atrial incision and the graft angle. The 3D heart model was beneficial in determining the position of the graft to be placed intracardially, the line of the atrial incision, and the location of the anastomosis to the pulmonary artery. In Case #19, the relationship among the inferior vena cava, hepatic vein, and pulmonary vein was complex and intricate, and it was challenging to construct the intracardiac route without simulation using the super-flexible 3D heart models. In the Fontan procedure, particularly in the univentricular heart associated with isomerism, the 3D heart model was useful in determining the intra-atrial route.

In contrast, the Norwood procedure for HLHS, which anastomoses the hypoplastic ascending aorta to the main pulmonary artery to create a neoaorta, can be planned using conventional imaging modalities such as echocardiography or cardiac CT. In this clinical trial, only one in four cases was considered “essential” and the remaining three cases were “very useful”. The super-flexible 3D heart models would be beneficial when surgeons plan special modifications of the Norwood procedure such as “chimney reconstruction” or the “interdigital arch reconstruction” [41].

No case or anatomic site was rated “misleading” in this clinical study. Two cases, Case #11 with TGA and Case #14 with HLHS, were rated “not useful” postoperatively by the primary surgeon. In Case #11, the surgeon decided to close the muscular VSD after the simulation, and the closure was successful. In Case #14, the primary surgeon commented that the autologous pericardium is expected to reconstruct a new aorta without stenosis when the distal portion is trimmed to have an obtuse angle. The efficacy assessment committee reviewed the scoring process for the two cases and determined that the pieces of evidence did not meet the criteria for “not useful” rating and were rated as “very useful”. 

### 4.3. Contribution of 3D-Printed Heart Models to Medical Education, Clinical Practice, and Development of New Operations

In the treatment of CHD, the small size of the heart, the complicated 3D anatomy, and the variety of diseases make accumulated experience essential for accurate and safe surgery. The basic types of cardiac surgery were recently replaced by advances in catheter-based interventions, such as device closure of atrial septal defect (ASD), valvuloplasty of the pulmonary or aortic valves, and angioplasty with balloon dilatation or stent implantation. For such simple and basic CHD, the training opportunities for pediatric cardiac surgeons are generally decreasing. Hence, performing accurate surgery for more complex CHD will be challenging and the need for patient-specific and super-flexible 3D heart models will continue to increase [42,43,44]. They will be used to transfer skilled surgical techniques to young surgeons [8,9,10,11,12,45,46], as well as catheterization techniques to young pediatricians [47] and anatomical information to medical students [48,49]. Another important application of 3D printing is to help develop new surgical procedures for complex CHD. A new surgical procedure was reported for truncus arteriosus repair using partial heart transplantation [50]

It is difficult to provide direct clinical evidence that 3D-printed heart models improve the surgical outcomes for CHD patients. Instead, this was indirectly demonstrated by the reduced time required for repeated simulation. Specifically, cardiac surgeons with varying levels of experience performed simulated surgeries twice and the simulation time was measured and evaluated. After repeated simulations, it was shown that the time required for the second simulated surgery was significantly reduced compared to the first [34,35,36].

Meanwhile, the efficacy of 3D-printed heart models was qualitatively validated in the clinical setting. In a clinical trial at 10 institutions in Europe and North America, the rate of surgical modification based on preoperative simulation using a 3D model was high, at 19 out of 40 cases [37]. In eight cases of planned biventricular repair, the surgical procedure was partially modified according to the preoperative simulation, and the treatment was improved. In four borderline cases where conservative treatment or functionally univentricular repair was initially planned, biventricular repair was successfully performed using preoperative simulation. The efficacy of 3D printing for CHD surgery was also demonstrated by several clinical and quantitative markers, including significant reductions in intensive care unit and mechanical ventilation time [51], as well as reduced, but not significant, 30-day readmission and mortality rates [52].

The importance of 3D printing is also evidenced by the fact that some medical societies have issued guidelines. The 3D Printing Special Interest Group of the Radiological Society of North America recently published guidelines on medical 3D printing and its appropriateness for clinical scenarios, including CHD [53]. In this guideline, they defined the appropriate use rating for clinical scenarios on a scale of one to nine. The congenital heart diseases that are considered “usually appropriate (score 7–9)” are ASD with anatomical complexity, VSD with additional complexity, unbalanced AVSD, IAA with LV outflow obstruction, persistent truncus arteriosus, MAPCA, isomerism with cardiac anomalies, TGA with PS, DORV, and atrioventricular and/or ventriculoarterial discordance. After the completion of the clinical trial, the Japanese Society of Pediatric Cardiology and Cardiac Surgery also published a similar guideline in 2022 for target diseases of the super-flexible 3D heart models in collaboration with our group.

### 4.4. Limitations and Future Directions

The super-flexible 3D heart models cost USD 2000 to produce, including polyurethane materials, other consumables, technical labor, software licenses, etc., and take 8.8 ± 3.4 days, which is expensive and time-consuming for practical use compared with other low-cost models [30,54,55,56]. The primary reason is that our project requires two molds made via stereolithography. To reduce the production time and cost, we recently developed an original UV-based inkjet 3D printing machine exclusively for manufacturing super-flexible 3D heart models (unpublished). It will improve the production time.

Currently, the cost of producing 3D-printed heart models is mainly covered by hospital expenses or departmental research funds, which is a major barrier to its integration into clinical practice. After receiving approval as an authorized medical device in Japan (30500BZX00177000), we applied for reimbursement from the National Health Insurance to cover the cost. This will improve the costs of production.

Recently developed 3D holograms with a stereoscopic view provide realistic 3D images and may be an excellent alternative to 3D heart models for a better understanding of CHD anatomy [57,58]. The application of virtual, augmented, and mixed reality with digital twin technology is also a promising educational tool for surgical procedures and mass healthcare training [59,60]. Such stereoscopic 3D images are visually powerful and comprehensive, but simulated surgery such as handling, retraction, cutting, and suturing can only be achieved with the accurate reproduction of the tangible and super-flexible 3D heart models.

Although high-fidelity simulation surgery can be performed with super-flexible 3D heart models, predicting and comparing hemodynamic changes after different surgical procedures in complex CHD is challenging. Four-dimensional flow MRI has emerged as a non-invasive imaging technique to visualize and quantify blood flow in the heart and vessels. By applying this technique, computational fluid dynamics (CFD) modeling can support the preoperative surgical decision-making process regarding cardiac functional analysis [61,62,63,64]. As a possibility for future clinical application, a study is being considered to clarify the usefulness of a system that combines the morphological diagnosis of the super-flexible 3D heart models with the hemodynamic prediction of the heart simulator. Recently, a unique multi-scale, multi-physics heart simulator, “ped UT-Heart”, was developed that can faithfully reproduce a patient’s heart in silico based on molecular and cellular functions using a supercomputer-based simulation model [65,66]. Unlike blood flow analysis using four-dimensional MRI, where the analysis is limited to a portion of the great vessels, the boundary conditions of “ped UT-Heart” are determined as a result of interactions with the contraction of the heart, the systemic circulation, and the pulmonary circulation. With this heart simulator, various surgical treatments can be tested on the computer, and the surgical procedure best suited to the patient’s hemodynamics can be selected and determined to provide customized medical treatment for each patient. Another potential application of 3D-printed heart models is the preservation of libraries of rare and precious heart specimens that have deteriorated over the years [67,68]. In collaboration with the Japanese Society of Pediatric Cardiology and Cardiac Surgery and Tokyo Women’s Medical University, we initiated a digital archiving project for autopsied specimens of CHD. As part of this project, typical teaching specimens from the CHD autopsy library will be CT-scanned, 3D-printed, and distributed to medical staff.

In the near future, the fusion of evolving technologies of anatomical and functional complexity, such as 3D printing, virtual reality, augmented reality, 3D and 4D holograms, and computational fluid dynamics, combined with artificial intelligence, would support and improve the outcome of surgery for complex congenital heart disease [69,70,71,72].

## 5. Conclusions

The super-flexible 3D heart models developed by us are patient-specific in size and anatomy and allow surgeons to simulate complex CHDs before surgery. The multicenter clinical trial demonstrated that super-flexible 3D heart models are useful for diagnosis and patient-specific surgical decisions. Therefore, the super-flexible 3D heart models have been approved as an authorized medical device and will be widely used for education, informed consent, and a better understanding of anatomy and simulation surgery to improve the prognosis of patients with complex CHD.

## Figures and Tables

**Table 1:** Demographics of the 19 patients.

Case	Sex	Age at Surgery	BW (kg)	Main Diagnosis	Additional Diagnosis	Past Intervention
1	M	9 m	6.6	TOF	severe RVOTS	–
2	F	1 y 1 m	7.5	TOF	severe RVOTS	BTS
3	M	10 m	8.3	PA/VSD	MAPCA	UF, BTS
4	F	6 y 8 m	18.7	critical PS	hypoplastic RV	BVP, RVOTR
5	M	27 d	3.4	DORV	subpulmonary VSD, CoA	–
6	M	2 m	3.9	DORV	subpulmonary VSD	–
7	M	4 m	9.0	DORV	non-committed VSD, severe PS, CAVC, MAPCA	UF, BTS
8	F	6 m	4.6	DORV	non-committed VSD, IAA	bilateral PAB, Norwood
9	F	1 y 2 m	7.0	DORV	non-committed VSD, PA atresia	bilateral PAB, RV-PA shunt
10	F	1 y 6 m	8.4	DORV	RIH, PS, large VSD, TAPVR	TAPVR repair, PAB
11	F	9 m	7.6	TGA (II)	multiple muscular VSD	PAB, BTS
12	M	1 y 3 m	8.9	TGA (III)	severe PS, ASD, PDA	PDA ligation, BTS
13	M	4 m	3.6	HLHS	(esophageal atresia)	bilateral PAB, PMI (repair of EA)
14	F	2 m	4.0	HLHS	–	bilateral PAB
15	M	3 m	4.1	HLHS	–	bilateral PAB
16	F	3 m	2.7	HLHS	–	bilateral PAB, BTS, BAS
17	M	1 y 11 m	12.8	UVH	RIH, PA, TAPVR	BTS, BDG
18	F	1 y 11 m	12.2	UVH	LIH, DORV, TAPVR	PAB, TCPS, DKS, CAVV repair
19	M	3 y 3 m	13.2	UVH	LIH, PA, TAPVR	BTS, TCPS CAVV repair

M: male, F: female, BW: body weight, TOF: tetralogy of Fallot, PA/VSD: pulmonary atresia with ventricular septal defect, , DORV: double outlet right ventricle, TGA: transposition of the great arteries, HLHS: hypoplastic left heart syndrome, UVH: functionally univentricular heart, RVOTS: right ventricular outflow stenosis, MAPCA: major aorto-pulmonary collateral arteries, RV: right ventricle, CoA: coarctation of the aorta, PS: pulmonary stenosis, CAVC: common atrioventricular canal, IAA: interrupted aortic arch, RIH: right isomeric heart, LIH: left isomeric heart, TAPVR: total anomalous pulmonary venous return, BTS: Blalock–Taussig shunt, BVP: balloon valvuloplasty, RVOTR: right ventricular outflow reconstruction, UF: unifocalization, PAB: pulmonary artery banding, PMI: pacemaker implantation, EA: esophageal atresia, BDG: bidirectional Glenn anastomosis, TCPS: total cavo-pulmonary shunt, DKS: Damus–Kaye–Stansel anastomosis, CAVV: common atrioventricular valve.

**Table 2:** Scheduled and actual surgery, overall evaluation, and one-year follow-up of the 19 patients.

Case	Main Diagnosis	Scheduled Procedures After Cardiac CT	Changes After Simulation	Changes at Surgery	LS by the Primary Surgeons	LS by the Assessment Committee	Unscheduled Intervention (<1 Year After Surgery)	Prognosis (1 Year)
1	TOF	TOF repair, PV sparing			Essential	Very useful		Alive
2	TOF	TOF repair (RV incision)	Valve sparing	RV non-incision	Very useful	Useful		Alive
3	PA/VSD	Rastelli, PA plasty			Essential	Essential		Alive
4	PA/IVS	Rastelli, RV overhaul			Very useful	Useful		Alive
5	DORV	ASO, CoA repair, IVR			Essential	Essential	Ao arch repair (44 days)	Alive
6	DORV	ASO, IVR			Essential	Essential		Alive
7	DORV	ND	BDG		Essential	Essential		Alive
8	DORV	BDG			Essential	Very useful		Alive
9	DORV	IVR, RVOTR, RV overhaul			Essential	Essential		Alive
10	DORV	Biventricular repair	BDG		Essential	Essential		Alive
11	TGA (II)	ASO (mVSD left open)	mVSD closure		Not useful	Very useful		Alive
12	TGA (III)	Half-turned truncal switch			Essential	Essential		Alive
13	HLHS	Norwood			Essential	Essential	TV plasty (59 days)	Dead (87 days)
14	HLHS	Norwood, TV plasty			Not useful	Very useful		Alive
15	HLHS	Norwood			Very useful	Very useful		Alive
16	HLHS	Norwood			Very useful	Very useful	Ao arch repair (6 days)	Dead (15 days)
17	UVH	TCPC			Essential	Essential	TCPC (348 days)	Alive
18	UVH	TCPC			Essential	Essential		Alive
19	UVH	TCPC			Essential	Essential	PVO relief (322 days)	Alive

Abbreviations are shown in Table 1. PV: pulmonary valve, ASO: arterial switch operation, BDG: bidirectional Glenn anastomosis, IVR: intraventricular rerouting, ND: not determined, mVSD: muscular VSD, TCPC: total cavo-pulmonary connection, LS: Likert scale, PVO: pulmonary venous obstruction.

**Table 3:** The results of the primary and secondary endpoints.

	Primary Endpoint	Secondary Endpoint		
	Primary Surgeons		Efficacy Assessment Committee	
“Essential” (n (%)) [95% CI]	13 (68.4%) [43.4–87.4%]		11 (57.9%) [33.5–79.7%]	
“Essential” + “Very useful” (n (%)) [95% CI]		17 (89.5%) [66.9–98.7%]		17 (89.5%) [66.9–98.7%]

## Data Availability

Restrictions apply to the availability of these data. Proprietary data were used, which may be available on reasonable request from the corresponding author.

## Supplementary Materials

The following supporting information can be downloaded at https://www.mdpi.com/article/10.3390/jcdd11120387/s1: Supplementary Figure S1: The average scores at each anatomical site. The anatomical scoring table quantified “essential” as a score of 4 and “misleading” as a score of 0. Video S1: Simulated Norwood procedure (chimney reconstruction of the aortic arch) in a 3-month-old infant with hypoplastic left heart syndrome (Case #15). Video S2: Simulated surgery of the half-turned truncal switch procedure in a 15-month-old infant with dextro-transposition of the great arteries and severe pulmonary stenosis (Case #12).
