# Supplementary figures and images for: Evaluation of the Efficacy and Accuracy of Super-Flexible Three-Dimensional Heart Models of Congenital Heart Disease Made via Stereolithography Printing and Vacuum Casting: A Multicenter Clinical Trial
Source: J Cardiovasc Dev Dis. 2024 Dec 3;11(12):387. doi: 10.3390/jcdd11120387 (PMC11677195; doi:10.3390/jcdd11120387)

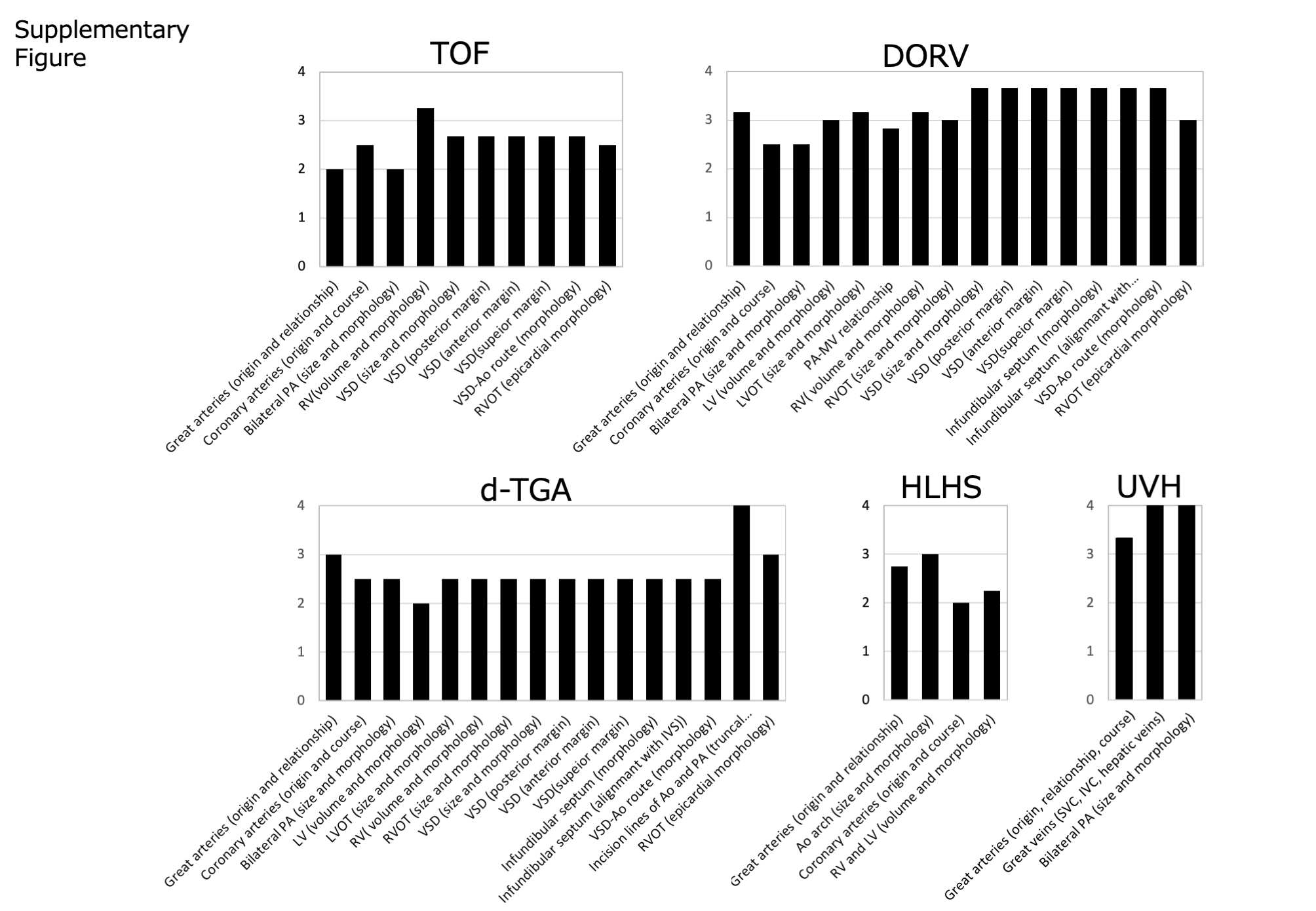

Supplement: Supplementary file 1 [file jcdd-11-00387-s001.zip › jcdd-3283316-supplementary.tiff]
